# Antiseizure adverse drug reaction and associated factors among epileptic patients at Jimma Medical Center: a prospective observational study

**DOI:** 10.1038/s41598-024-61393-9

**Published:** 2024-05-21

**Authors:** Yadeta Babu Bayane, Wakuma Wakene Jifar, Robera Demissie Berhanu, Dame Habtamu Rikitu

**Affiliations:** 1 Department of Clinical Pharmacy, Institute of Health Science, Wallaga University, Nekemte, Ethiopia; 2 Department of Pharmacy, College of Health Science, Mattu University, Metu, Ethiopia; 3 School of Nursing and Midwifery, Institute of Health Science, Wallaga University, Nekemte, Ethiopia; 4 Department of Obstetrics and Gynecology, College of Health Sciences, Addis Ababa University, Addis Ababa, Ethiopia

**Keywords:** Adverse drug reactions, Antiseizure drugs, Epilepsy, Ethiopia, Neuroscience, Neurology

## Abstract

A growing body of evidence suggests that adverse drug reactions (ADRs) are a major cause of morbidity and mortality in the healthcare system. Fifteen to twenty-five percent of patients with epilepsy discontinued antiseizure drugs (ASDs) within 6 months of therapy owing to intolerable adverse drug reactions. In Ethiopia, the prevalence of antiseizure adverse drug reactions and associated factors was not extensively conducted in advanced settings like Jimma Medical Centers. Hence, the objective of this study is to assess patterns of adverse drug reactions and associated factors among ambulatory epileptic patients at tertiary hospitals in Ethiopia. A hospital-based prospective observational study was spanned for 1 year. Two hundred ninety patients were consecutively recruited into the study from all epileptic patients attending the ambulatory clinic. Relevant data were collected through patient interviews and medical chart reviews. The causality assessment was done by using the Naranjo Probability Scale. Epi-Data manager version 4.6.0.4 was used for data entry and statistical analysis was performed by Statistical Package for Social Science version 25.0 (SPSS). Stepwise backward logistic regression analysis was done to identify factors that increase the risk of antiseizure adverse drug reactions. The mean (± SD) age of the participants were 29.91(± 11.26) years. The overall prevalence of ADR was 33.8% (95% CI 29.2–39.9%). A total of 110 adverse drug reactions were identified among 98 patients with an average of 1.12 per patient. ADRs were frequently reported with phenobarbital (52.04%) and phenytoin (34.70%). The commonly identified adverse drug reactions were epigastric pain (27.55%) and central nervous system drowsiness (23.46%). Comorbidity (AOR = 5.91, 95% CI (2.14–16.32), seizure-free period of fewer than 2 years (AOR = 1.94, 95% CI (1.18–3.19), and polytherapy (AOR = 1.35, 95% CI (1.80–2.26) were significantly associated with adverse drug reactions. This trial had a comparatively high percentage of adverse medication reactions. Adverse medication reactions were more common in patients with polytherapy, comorbidities, and seizure-free durations less than two years. Therefore, medical practitioners should advise patients who exhibit these traits on how to reduce or avoid bad drug responses or provide comfort in the event of small incidents.

## Introduction

Epilepsy is the syndrome of two or more unprovoked seizures that occur more than 24 h apart^1^. Epilepsy is associated with adverse psychosocial outcomes, disability, higher rates of psychiatric comorbidity, and approximately threefold increased mortality^2^. In Life time one in 26 people develop epilepsy, and it is the fourth most common neurological disorder in the world^3^. Worldwide there are seventy million people with epilepsy, and 90% of people with epilepsy live in low- and middle-income countries^4,5^. Epileptic seizure has a lot of negative consequences on the patient’s psychology and social life such as education, relationships, and employment status^6^.

Antiseizure medications are standard treatment modalities with the primary goal of achieving seizure freedom ideally without adverse events, reducing morbidity, mortality, and seizure-related accidents, and improving quality of life^7,7^. In 70% of the patients with epilepsy, these goals are feasible with the optimum use of antiseizure drugs (ASDs)^8^. The centrally acting drugs like antiseizure, contribute to adverse drug reactions (ADRs) such as insomnia, sedation, increasing suicidal tendencies, and depression^6^. These ADRs compromise the benefits of ASDs^6^. The World Health Organization (WHO) defines adverse drug reaction (ADR) as “any noxious, unintended, or undesired effect of a drug that occurs at doses used in humans for prophylaxis, diagnosis, investigation, therapy, or for the adjustment of physiological function^9,10^.

ADR has long been a problem in the health industry and is a big worry in the healthcare system. ADR has impacted most individuals throughout medical history, significantly increased morbidity and mortality, and placed a heavy demand on healthcare resources^11^. Because of its potentially serious consequences, adverse drug reactions (ADRs) may have a significant impact on clinical practice and the economy. ADRs is either avoidable or unavoidable and it may resulted in transient or permanently debilitating effects, including death and financial costs for the patients, healthcare institutions and society as a whole^12^.

ADRs are a significant public health problem in the world. Not only do ADRs cause death and injury but they also affect the length of stay in hospitals which in turn leads to increased healthcare costs and decreased patient productivity^13^. In the United States (US) 44,000 to 98,000 deaths occur annually from medication errors. Of this total, an estimated 7000 deaths occur due to ADRs^13^.ADRs account for 4.2–30% of hospital admissions in Canada, 2.5–10.6% of admissions in Europe, 5.7–18.8% of admissions in Australia^14^, and 6.2% of all admissions in Southern Italy^15^. In Norway, 19.7% of patients had drug-related emergency visits and ADRs were the most common causes of these visits^16^.

The causes of adverse drug reactions are related to many factors including drug-related, patient-related, disease related, social, and adverse drug-related factors^11,17^. The patient’s physiological and illness status influence unfavorable drug response; very young and elderly patients are more vulnerable to an unfavorable drug response than adult patients. This is usually due to a significant difference in metabolism and excretion pattern at this level and the decreased functional reserve in the extremities of age^17^.

Studies showed that 22–31% of patients with epilepsy (PWE) developed antiseizure-associated ADR^18–20^. Adverse drug reactions of ASDs are a leading cause of treatment failure among people with epilepsy^19^. Fifteen to twenty five percents of patients with epilepsy discontinued ASDs within 6 months of therapy owing to intolerable adverse drug reactions^20,21^. The previous study across various settings identified factors associated with ADRs among people with epilepsy. These are polytherapy, age, alcohol intake, frequent seizure, gender, and pretreatment seizure numbers^13,18,21,22^. Despite these potentially modifiable factors related to ADR among PWE, no prior extensive study has been conducted in the current study setting. Therefore, this prospective observational study was designed to carried out the prevalence of ADR and its associated factors among patients with epilepsy at the ambulatory clinic of Jimma medical center (JMC).

## Materials and methods

### Study design, area, and period

A prospective observational study design was spanned from December 2020 to November 2021 at the ambulatory clinic of Jimma Medical Center (JMC). JMC is a referral and teaching governmental institution located in Jimma town of Oromia Regional State, Ethiopia. Currently, the center provides services for 20 million people from the Jimma zone and is a referral center for South Western part of Ethiopia.

### Inclusion and exclusions criteria

Age of ≥ 18 years, on ASDs for a minimum of 1 year, and agreeing to give informed consent were inclusion criteria.

Patients with unstable psychiatric illness, not adhere to their next follow-up schedule, and incomplete medical records were excluded from the study.

### Sample size and sampling technique

The sample size was calculated by using a single population proportion formula by considering the proportion of ADR, P = 0.22^18^, n = the desired sample size, Z = level of significance at 95% confidence interval which is 1.96, d = margin of error which is 0.05,

$${\text{n }} = \frac{{{\text{z}}^{{2}} {\text{p}}\,\,\left( {{1} - {\text{p}}} \right)}}{{{\text{d}}^{{2}} }} = \, \frac{{({1}.{96})^{{2}} \,0.{22}\,\,\left( {{1} - 0.{22}} \right)}}{{\left( {0.0{5}} \right)^{{2}} }} = { 264}$$

By adding a 10% non-response rate, the final sample size of 290 was calculated. A consecutive sampling method was used to include study participants.

### Data collection tools and procedures

Data were collected through the patient interview and medication chart review. The data collection tools were prepared after reviewing relevant kinds of literature and modified to address the objectives of this study^9,23–26^. Patients were interviewed after leaving the physician’s office for medication refills. Information related to the ADR was collected through the patient interview and augmented by the review of the patient’s medical records. The Causal relationship between ADR and treatment was assessed by a researcher, with the Naranjo Algorithm^24^. Naranjo ADR probability scale is validated for the assessment of ADRs and produced the most consistent results^27–29^. The Naranjo Algorithm consists of 10 questions that are answered as either Yes, No, or “Do not know”. Different point values (− 1, 0, + 1, or + 2) are assigned to each answer^24^. Total scores range from − 4 to + 13; the reaction is considered definite if the score is 9 or higher, probable if 5 to 8, possible if 1 to 4, and doubtful if 0 or less^24^. Medication belief is assessed by belief about medication questionnaires (BMQ)^26^. Belief about Medications Questionnaire (BMQ) is a validated tool to assess the beliefs of patients about their medications^30^. This is 10 items self-reported Likert scale, which are further categorized as the medication necessity scale and medication concern scale, each containing five items. The patient’s belief was considered to be positive when the average sum of the 5-item patient’s medication necessity scale score exceeded the average 5-item medication concern scale, if not it was considered negative^5^. Three pharmacists, a bachelor of degree holders were participated in the overall data collection processes.

### Data processing & analysis

The collected data were entered into Epidata Manager version 4.6.0.4 and then exported to SPSS version 25.0. A descriptive statistic was calculated for dependent and independent variables. To select candidate variables for multivariable analysis a bivariable logistic regression analysis was done and all variables with a p-value of < 0.25 were taken to multivariable analysis. A multivariable logistic regression analysis was performed to identify independent variables associated with ADR. From multivariable outputs variables with a *p*-value of < 0.05 were considered as statistically significant variables associated with ADR. A 95% confidence interval (CI) was used to assess the strength of association between the independent and the primary outcome of the variable.

### Ethical considerations

The approval letter for this study was taken from the institutional review board (IRB) of Jimma University, College of Health Sciences. The objective of the study was explained and written informed consent was obtained from study participants. For the matter of patient confidentiality, the name and addresses of the patients were not included in the data collection tools. All methods were performed in accordance with relevant guidelines and regulations.

## Results

### Socio-demographic characteristics of study participants

A total of 310 patients were approached, eight patients were excluded from analysis due to incomplete medical records, five patients were excluded for instability of the illness, and seven patients were excluded for lost from follow-up. Of 290 studied patients, 54.48% were male, and 46.55% were within the age range of 18–25 years. About 27.93% had no formal education and only 12.76% of participants were employed (Table 1).

**Table 1:** Demographic characteristics of study participants at the neurology clinic of JMC, 2021.

Variables	Frequency (n)	Percentage (%)
Age (years)		
18–25	135	46.55
26–44	116	40.00
+ 45	39	13.45
Gender		
Male	158	54.48
Female	132	45.52
Educational status		
No formal education	81	27.93
Formal education	209	72.07
Occupation		
Unemployed	253	87.24
Employed	37	12.76

### Clinical characteristics of the patients with epilepsy

Half (50.69%) of the participants had a seizure duration of more than 10 years and about three fourth of them had been diagnosed at the age of older than 10 years. Two third (65.52%) of the study subjects had no seizure episode since the last visit, whereas (47.58%) of patients had a seizure-free period of fewer than 2 years. The majority (62.75%) of the patients have no comorbid medical condition and half the participants were taking single ASD (Table 2).

**Table 2:** Clinical characteristics of study participants at the neurology clinic of JMC, 2021.

Variables	Frequency	Percentage (%)
Age at diagnosis of seizure (years)		
Younger than or equal to 10 years	72	24.82
Older than 10 years	218	75.18
Seizure duration (years)		
Less than or equal to 10 years	143	49.31
Greater than 10 years	147	50.69
Seizure episodes since the last visit		
Yes	100	34.48
No	190	65.52
Seizure free period		
Less than 2 years	138	47.58
More than 2 years	152	52.42
Medication belief		
Positive	192	66.20
Negative	98	33.80
Comorbidities		
No	182	62.75
Yes	108	37.25
Number of ASD		
One	145	50.00
≥ Two	145	50.00

### Profile of adverse drug reactions among people with epilepsy

**Figure 1:**
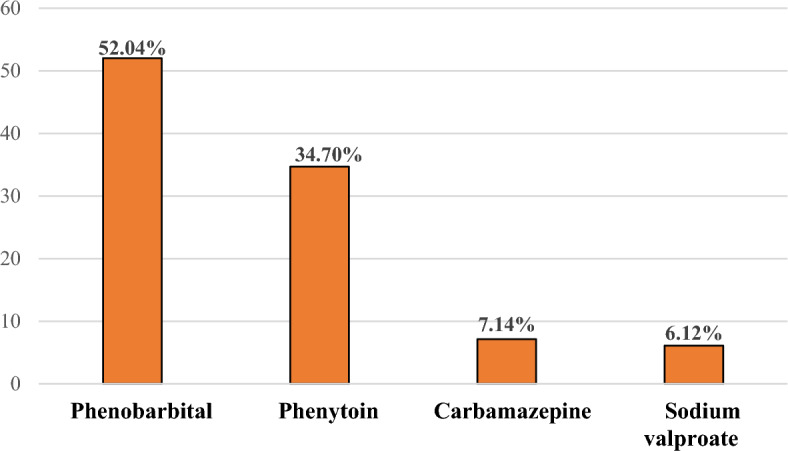
Antiseizure drugs associated with ADR at the neurology clinic of JMC 2021.

From 290 studied participants 98 were experienced at least one antiseizure associated ADR with an overall prevalence of 33.8% (95% CI 29.2–39.9%). The most commonly recorded ADRs were epigastric pain (27.55%) and central nervous system drowsiness (23.46%). According to the Naranjo probability scale of causality assessment in (52.72% and (39.09%) of the case the level of causality was possible and probable respectively. Patients counseling and decreasing the dose of ASD were the two common measures taken by pharmacists (Table 3). ADRs were frequently reported with the two most widely used ASDs:—phenobarbital (52.04%) and phenytoin (34.70%) (Fig. 1).

**Table 3:** Adverse drug reactions profile among epileptic patients at the neurology clinic of JMC 2021.

Variables	Frequency (n)	Percentage (%)
Adverse drug reactions		
Yes	98	33.80
No	192	66.20
Types of adverse drug reactions		
Epigastric pain	27	27.55
Drowsiness	23	23.46
Headache	20	20.40
Confusion	11	11.22
Gum hypertrophy	10	10.20
Rash	6	6.12
Tremor	4	4.08
Others^a^	9	9.18
Level of causality assessment		
Probable	43	39.09
Possible	58	52.72
Doubtful	9	8.18
Measurement taken		
Patient counseling	52	47.27
Decreasing the dose	39	35.45
Switching of medication	19	17.27

^a^Loss of hair, irritability, weakness, dizziness, and insomnia.

### Factors associated with ADR among patients with epilepsy

The results of multivariable analysis output showed that patients who had experienced seizure episodes in the last 2 years were 1.94 times more likely to have ADR as compared to patients without seizure episodes (AOR = 1.94, 95% CI (1.18–3.19)), patients with one or more comorbidity were 5.91 times more likely to develop ADR as compared to patients with no comorbid medical conditions (AOR = 5.91, 95% CI (2.14–16.32)). Additionally, those patients who were taking two or more ASD were 1.35 times more likely to have ADR as compared to patients who were on monotherapy (AOR = 1.35, 95% CI (1.80–2.26)) (Table 4).

**Table 4:** Multivariable logistic regression analysis of factors associated with adverse drug reactions among epileptics at JMC, 2021.

Variables	ADR		Bivariate analysis		Multivariate analysis	
Age (years)	Yes	No	COR	P-value	AOR	P-value
18–25	41	94	1			
26–44	44	72	0.85 (0.51–1.34)	0.440	–	–
+ 45	14	25	1.23 (0.23–6.51)	0.806	–	–
Gender						
Male	52	106	1			
Female	48	84	0.87 (0.54–1.38)	0.554	–	–
Educational status						
No formal education	27	54	1.20 (0.38–3.72)	0.134	1.06 (0.63–1.78)	0.806
Formal education	71	138	1		1	
Occupation						
Unemployed	85	168	1.06 (0.43–2.06)	0.899	–	–
Employed	13	24	1			
Age at diagnosis						
Less than or equal 10 years	23	49	1.11 (0.65–1.91)	0.184	0.85 (0.55–1.82)	0.997
Older than 10 years	75	143	1		1	
Seizure duration (years)						
Less than or equal 10 years	46	97	1			
More than 10 years	53	94	0.88 (0.55–1.39)	0.586	–	–
Seizure episodes since last						
Yes	45	55	2.21 (1.36–3.57)	0.001	1.33 (0.66–2.70)	0.420
No	49	141	1		1	
Seizure free period						
Less than 2 years	61	77	2.42 (1.51–3.89)	< 0.001	1.94 (1.18–3.19)	**0.009**
More than 2 years	34	118	1		1	
Medication belief						
Positive	52	140	1		1	
Negative	43	55	1.93 (1.19–3.13)	0.007	1.10 (0.62–1.97)	0.729
Comorbidities						
No	41	141	1		1	
Yes	46	62	2.41 (1.44–4.03)	0.001	2.11 (1.25–3.57)	**0.005**
Number of ASD						
One	37	108	1		1	
≥ Two	57	88	1.85 (1.59–2.95)	0.01	1.35 (1.80–2.26)	**0.025**

## Discussion

The overall aim of treatment in patients with epilepsy (PWE) is complete seizure freedom with little to no medication-related side effects^31^. However, the prolonged use of ASD is associated with various idiosyncratic reactions and clinically significant drug interactions, that may result in treatment letdown in about 40% of patients^19^. In this finding, ADRs were found in 98 (33.8%) of PWE, and most commonly occurred within the age category of 26–44 years. This finding was relatively similar to the study reported from Bishoftu general hospital^32^, and contrary to the results from various settings including India, Italy, and Hyderabad where the prevalence of ADR were 3.07%, 3.2%,4.67% respectively^15,33,34^. This variation might be due to the special considerations taken by the practicing physicians regarding the prescribing and titrating doses of ASD, and the availability of new ASD in the former settings.

Regards to the distribution of ADR, epigastric pain (27.55%), drowsiness (23.46%), and headache (20.40%) were the most commonly reported ADR. This is in line with the study conducted at the neurology clinic of UoGRH, Northwest Ethiopia, and Amanuel Specialized Mental Hospital (ASMH)^18,35^. In contrast to our study, headache, and Loss of appetite were the most commonest ADRs recorded in Bishoftu general hospital and Tertiary Care Hospital of India^32,33^.

From the total of 98 reported ADRs, phenobarbital was the primary cause (52.04%) followed by phenytoin (34.70%) and carbamazepine (7.14%). This finding was augmented by the prior reports from (ASMH) and UoGRH^25,35^, but it is in contrast to the other finding from Tertiary Care Hospital of India, where 42.5% of ADR were related to valproate^33^. The probable justifications for this disagreement could be in resource-poor settings like Ethiopia it is old-generation ASDs like phenobarbital and phenytoin that were frequently used in the management of epilepsy^5^.

Studies showed that 30–40% of patients with epilepsy suffered from uncontrolled seizures on a single antiseizure drug(ASD)^36^. For these patients, combination therapy is standard of practice rather than optional^37^. The combined use of ASD is usually not without problems. Previous studies established that polytherapy leads to a higher risk of adverse drug effects compared with monotherapy^19,22,38^. Similarly, in this study patients undergoing polytherapy tended 1.35 times (AOR: 1.35, 95% CI 1.80–2.26) at increased risk of ADR as compared to monotherapy. This finding is potentiated by the other study conducted at the Northwest Ethiopia and Wangaya Regional Hospital in Denpasar^25,39^. This is probably because of overlapping effects and drug interactions ADR increases as the number of co-administered ASD increases.

There is increasing recognition that epilepsy can be associated with a broad spectrum of comorbidities^40^. Several diseases, including depression, anxiety, dementia, migraine, heart disease, peptic ulcers, and arthritis are up to eight times more common in people with epilepsy than in the general population^41^. It is known that comorbidity is strongly associated with a higher risk of ADR^15,42^. In our finding also the risk of experiencing ADR among epileptic patients with one or more comorbidities was increased by 5.91 times (AOR: 5.91, 95% CI 2.14–16.32) as compared with no comorbid medical conditions. The probable justifications for this factor could be multiple disease conditions influence susceptibility to ADRs and predispose patients to ADRs due to the concurrent use of many medicines^13,43^.

Adverse drug reactions can contribute to treatment failure in up to 40% of patients and can affect the ultimate quality of life independent of seizure control^44^. In the present study, seizure-free periods of fewer than two years were significantly associated with ADR (AOR: 1.94, 95% CI 1.18–3.19). This finding is supported by the various literature across the globe^5,7,45^. The probable justification for this factor may be due to the negative effect of ADR on adherence that affects the seizure control status.

### Strength and limitation

The quality of the data given in this study is significantly higher than in other studies because it is a prospective observational study. Patients' replies are used in the assessment of ADR, medicine belief, and Naranjo casual assessment, which may skew the results or alter their genuine value. Furthermore, because this study was only done in one location, it might not be very generalizable.

## Conclusions

This study looks into a larger percentage of ADR than other national and international studies that have been done. ADR risk factors included polytherapy, comorbidities, and a seizure-free duration of less than two years. Healthcare providers who focus on patients with the aforementioned issues may be able to address or reduce the risk of ADR.

## Data Availability

All the data used in the current study are available from corresponding author upon the reasonable request.
